# Systematic Review of Contact Investigation Costs for Tuberculosis, United States

**DOI:** 10.3201/eid3107.241827

**Published:** 2025-07

**Authors:** Garrett R. Beeler Asay, Kai H. Young, Tempest D. Hill, Gibril J. Njie

**Affiliations:** Centers for Disease Control and Prevention, Atlanta, Georgia, USA

**Keywords:** tuberculosis, health policy, health economics, bacteria, respiratory infections, tuberculosis and other mycobacteria, United States

## Abstract

Contact investigation is a fundamental component of tuberculosis (TB) programs that drives prompt diagnosis and treatment of *Mycobacterium tuberculosis* infection among those exposed. Few studies have examined contact investigation costs for TB. We conducted a systematic review of TB contact investigation costs in the United States by searching English-language articles published during January 1990–August 2024 in electronic databases, including MEDLINE, Embase, CINAHL, and Scopus. We identified 2,920 titles and abstracts; 10 studies met our inclusion criteria. We abstracted costs for labor, diagnostic tests, and chest radiographs. Labor cost per contact was estimated at $175.94 (range $79.97–$293.51); total cost, including diagnostic testing and chest radiography, was $228.93 (range $132.95–$346.49).The overall cost of contact investigation in the United States was $9.94 (range $5.77–$15.04) million in 2022; total cost during 2013–2022 was $137.35 million. Contact investigations are essential to prevent TB and avert TB-related labor and diagnostic costs.

Tuberculosis (TB) is a leading cause of infectious disease deaths globally; an estimated 1.1 million deaths occurred in 2022 (*1*). The United States is considered a low TB-incidence country by the World Health Organization, having made great strides toward reducing TB incidence since 1993 (*2*). During 1993–2020, the annual number of TB cases in the United States declined by 64%, from 25,102 to 8,920 cases (*3*). However, after the COVID-19 pandemic resulted in global health disruptions in 2020, TB cases have increased each year; an increase of 16% during 2022–2023 has been reported (*4*).

Contact investigation is a critical activity conducted by public health departments to interrupt infectious disease transmission (*5*). Contact investigation incorporates case finding and classification, case interviews to identify contacts, evaluation and testing of high-risk contacts for the presence of *Mycobacterium tuberculosis* by using either the Mantoux tuberculin skin test (TST) or an interferon-γ release assay (IGRA), and establishing a system for tracking persons exposed to *M. tuberculosis* (*6*). In addition, TB programs provide treatment to persons who have either TB infection or disease (*5*,*7*). Contact investigation prevents TB transmission and, thereby, future TB cases and costs; 1 analysis estimated that during a 10-year period, outbreak investigations could avert 5,560 TB cases and $102 million in healthcare costs (*8*).

We conducted a systematic literature review to quantify the labor cost and resources needed to conduct TB contact investigations in US settings. Moreover, to estimate national total TB contact investigation costs over a 10-year period (2013–2022), we combined labor cost estimates from the systematic review with TB contact investigation data reported to the Centers for Disease Control and Prevention (CDC) and TB testing data from a privately insured population.

## Methods

### Evidence Acquisition

A multidisciplinary team consisting of TB scientists (T.H. and K.H.Y.), a health economist (G.R.B.A.), and a systematic review methodologist (G.J.N.) from the CDC’s Division of Tuberculosis Elimination, National Center for HIV, Viral Hepatitis, STD, and TB Prevention, convened to conduct this systematic review. We used established economic evaluation methods adapted from the Guide to Community Preventive Services (*9*,*10*). We sought to answer the following research question: what are the per contact costs of TB contact investigations from a health system perspective? We included costs for personnel, materials, or supplies related to contact investigation; laboratory and diagnostic testing; medications; transportation; public relations; and communications in the analysis.

### Search Strategy and Inclusion Criteria

**Figure 1:**
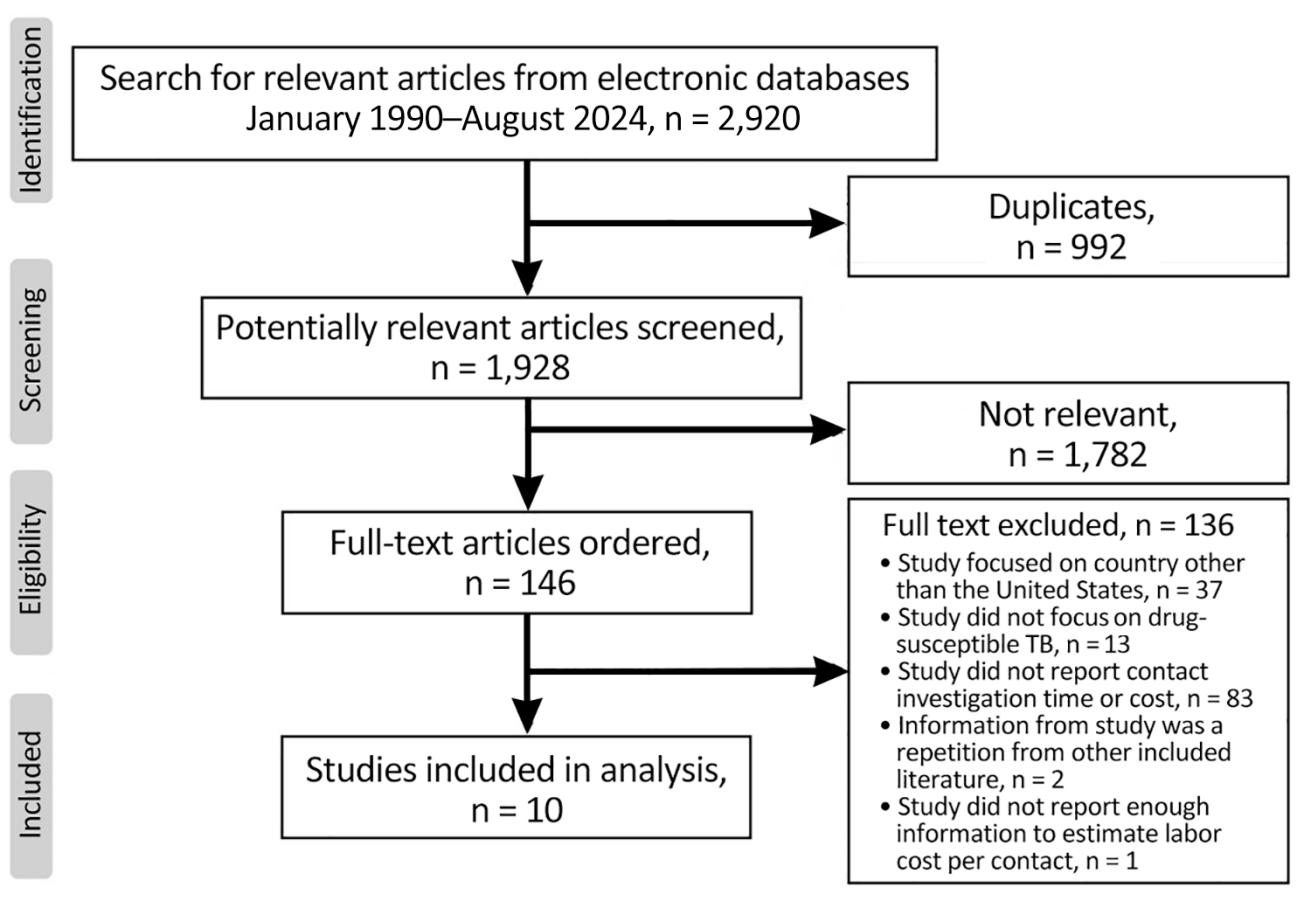
Flow diagram of studies identified, included, and excluded in systematic review of contact investigation costs for tuberculosis, United States. Diagram was generated from the Preferred Reporting Items for Systematic reviews and Meta-Analyses reporting guidelines template (https://www.prisma-statement.org).

We consulted a librarian to search for published studies that evaluated the cost of TB outbreak and contact investigations. We searched electronic databases, including MEDLINE, Embase, CINAHL, and Scopus, for English-language articles published during January 1990–August 2024 (Figure 1). We used the following medical subject headings: “tuberculosis” OR “latent tuberculosis”; search terms were synonyms of “outbreak investigation,” “epidemiological investigation,” “contact investigation,” AND “cost”; and “economic,” “expense,” “expenditure,” AND “United States.” We excluded articles if the study was not conducted in the United States, did not focus on drug-susceptible TB, or did not include contact investigation time or labor cost; if the cost information within the study was repeated from an earlier study; or if the study did not provide enough information to estimate labor cost per contact.

### Cost Data Abstraction

We abstracted data on study and participant characteristics, including the setting, outbreak size, contact investigation strategy, and demographic information. Using the ingredients method (*11*), in which program costs are estimated by adding each component of cost, we abstracted direct program cost components for personnel, laboratory and diagnostic procedures, labor cost associated with contact investigation, public relations and communications, and other administrative costs. We included costs of chest radiographs (but not the cost of follow-up visits), directly observed therapy, and latent TB infection treatment, because those costs were associated more with treatment rather than with contact investigation. Any disagreement on data abstraction elements between reviewers (G.R.B.A. and G.N.J.) was resolved by consensus or by a third reviewer (K.H.Y.). For each included study, we assigned a quality rating according to criteria developed for The Community Guide (*12*). We rated studies as very good, good, satisfactory, or unsatisfactory; we excluded studies rated as unsatisfactory from the analysis.

### Contact Investigation Labor Cost per Contact

For studies that reported person-hours required to conduct a contact investigation, we converted the reported person-hours to a monetary value by using region-specific wage data for a registered nurse according to the US Bureau of Labor Statistics (*13*). We estimated total compensation for labor by inflating wages by 30% to account for fringe benefits (*14*). No studies reported patient costs associated with contact investigation. We converted reported cost per case to cost per contact by dividing cost per case by the number of contacts per case. Although 2 studies reported costs of contact investigation for unconfirmed cases, we only reported mean contact investigation costs for confirmed cases, because sources did not report the number of contacts for unconfirmed cases, and some suspected cases were later identified to be non-TB cases. If a study reported labor costs that included TST costs, we used the TST cost value reported in the study. Otherwise, if the study did not report a cost for TST, we subtracted the Medicare reimbursement rate for TST costs in 2022 (*15*). We excluded reported surveillance and outbreak costs from the cost analyses because studies did not report costs in sufficient detail to determine those costs (*16*,*17*).

We updated all monetary values to 2022 US dollars by using the healthcare component of the Personal Consumption Expenditure Index from the Bureau of Economic Analysis (*18*). Because the cost data were limited in sample size and highly skewed, we used a nonparametric bootstrap to estimate the 95% CI by using the boot package in R version 4.4.1 (The R Project for Statistical Computing, https://www.r-project.org).

### National Cost of Contact Investigation

To estimate the 2022 US national costs of contact investigation and the total 10-year costs of contact investigation during 2013–2022, we obtained data on contact investigation activities reported to CDC through the Aggregate Reports for Program Evaluation Contact Investigation form submitted through the CDC’s National Tuberculosis Indicators Project web-based tool (*19*–*21*). In addition, we used aggregate report data on the number of contacts who tested positive for TB to estimate the percentage of contacts who might test positive and be referred for a chest radiograph (Appendix Table 1).

We used the MarketScan Truven Health commercially insured database (https://marketscan.truvenhealth.com/marketscanportal) to estimate the proportion of tests conducted during 2013–2022 according to paid claims for 3 diagnostic tests: TST, QuantiFERON-TB Gold (QFT) blood assay (QIAGEN, https://www.qiagen.com), and T-SPOT (Oxford Immunotec Ltd./Revvity, https://www.revvity.com) (*22*). Although some patients received >1 test, the analysis focused on claims for the first test (Appendix). For costs not reported by studies (e.g., cost of QFT and T-SPOT), we used published Medicare reimbursement rates (*15*,*23*). We assumed that all contacts received a test and that the tests were used at the same proportion as that in the MarketScan commercially insured population (Appendix). 

## Results

Our search strategy for this review identified 2,920 titles and abstracts. Of those, we determined 165 full-text articles were appropriate for review; 10 studies met the criteria for inclusion (Figure 1) (*7*,*16*,*17*,*24*–*30*). We judged all 10 studies as at least satisfactory quality, meeting the inclusion criteria (Appendix). Of the 10 included studies, we judged 6 were very good quality (*17*,*24*,*26*,*27*,*29*,*30*), 3 were good quality (*16*,*25*,*28*), and 1 was satisfactory quality (*7*).

More than half (60%) of the included studies were published after January 1, 2010 (*17*,*24*–*26*,*28*,*30*); 2 studies were published in the early 2000s (*7*,*29*) and 2 in the 1990s (*16*,*27*) (Table 1). Most included studies were from the South Census region (*17*,*25*,*28*,*29*); 3 studies were from the West region (*7*,*24*,*30*), and 1 study was from the Northeast region (*26*). No studies were included from the Midwest region. Moreover, we included 1 multistate study (*16*) and 1 study with an unknown geographic location (*27*). Most studies were community-focused (*7*,*16*,*17*,*28*,*29*); other settings were hospitals (*25*–*27*) and 1 high school (*24*). Most (70%) studies comprised cost analyses; 2 studies consisted of cost-effectiveness analyses (*7*,*29*). One study provided person-hours for personnel involved in TB contact investigations without costs (*24*).

**Table 1:** Studies included in systematic review of contact investigation costs for tuberculosis, United States

Characteristic	No. (%) studies, N = 10	References
Publication period		
1990s	2 (20)	(*16**,**27*)
2000s	2 (20)	(*7**,**29*)
2010s	5 (50)	(*17**,**25**–**28*)
2020s	1 (10)	(*30*)
Study location		
Northeast	1 (10)	(*26*)
Midwest	0 (0)	
South	4 (40)	(*17**,**25**,**28**,**29*)
West	3 (30)	(*7**,**24**,**30*)
Multistate	1 (10)	(*16*)
Other*	1 (10)	(*28**)*
Setting		
Community	6 (60)	(*7**,**16**,**17**,**28**–**30*)
Healthcare	3 (30)	(*25**–**27*)
School	1 (10)	(*24*)

*Unknown location.

### Contact Costs

**Figure 2:**
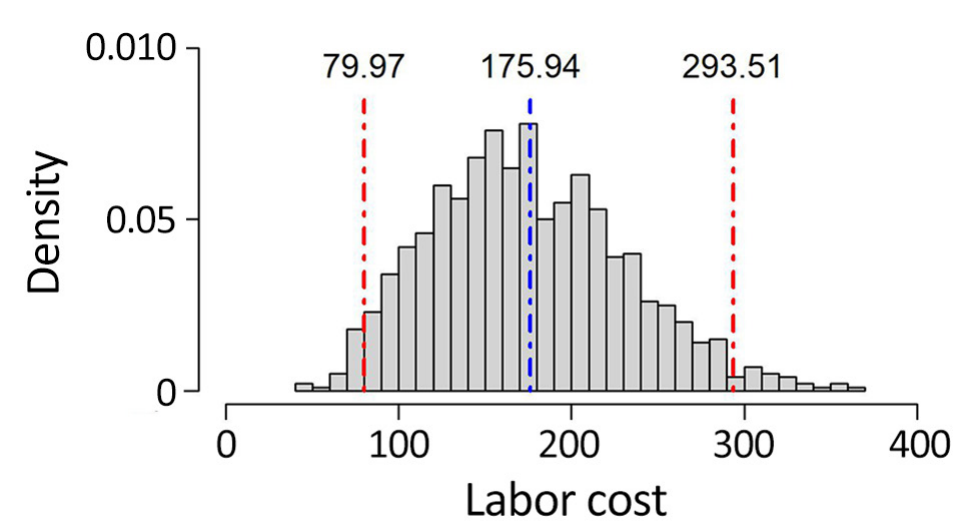
Density plot for labor costs of tuberculosis contact investigations in the United States. y-axis indicates bootstrapped probability density function (1,000 random sample draws). Costs per investigation were determined by using 2022 US dollar values. Blue dashed vertical line indicates the mean cost ($175.94); red dashed vertical lines indicate 95% CI ($79.96–$293.51).

We recorded outcomes for contact investigation costs and characteristics of the included studies (Table 2). Overall, the mean labor cost for contact investigation from the 10 studies was $175.94 (median $109.67) per contact in 2022 US dollars (*7*,*16*,*17*,*24*–*30*). The 95% nonparametric bootstrapped CI for the mean cost ($175.94) was $79.96–$293.51 (Figure 2). When stratified by setting, 6 studies from a community setting reported a mean labor cost of $251.98 (median $189.03) (*7*,*16*,*17*,*28*–*30*). Studies set in a hospital (n = 3) reported a lower mean labor cost of $72.95 (median $56.74) per contact (*25*–*27*), and 1 study conducted at a school reported a labor cost of $28.72 per contact (*24*). Among studies reporting cost per case, 3 were in a hospital setting (*25*–*27*) and 4 were in a community setting (*7*,*17*,*28*,*30*). Studies also reported costs per test for TST (n = 4; mean $15.95, median $14.59), cost of test follow-up (n = 4; mean $63.65, median $78.15), costs of chest radiographs per view (n = 2; mean and median $43.79), and surveillance and other outbreak-related costs per contact (n = 2; mean and median $33.34).

**Table 2:** Contact investigation costs for confirmed tuberculosis cases reported in systematic review, United States*

Reference	Setting	State(s)	No. cases investigated	No. contacts	Contact investigation costs, US $			
					Labor cost per contact	TST cost	Test follow-up and examination	Chest radiography
(*16*)	Community	AL, IL, NJ, TX, CA	26,283	NR	38.63	18.66	10.00	56.81
(*24*)†	School	CO	1	1,249	28.72	NR	NR	NR
(*25*)	Hospital	TX	59	880	114.18	NR	NR	NR
(*26*)†	Hospital	NY	34	1,394	56.74	NR	NR	NR
(*27*)	Hospital	NR	NR	81	47.94	14.99	88.30	NR
(*17*)	Community	TX	108	1,675	640.28	14.19	NR	NR
(*28*)	Community	NC	99	506	105.16	NR	NR	NR
(*29*)‡	Community	AL	NR	NR	349.73	NR	82.00	30.77
(*30*)	Community	CA	81	NR	235.77	NR	NR	NR
(*7*)	Community	CA	2,032	17,774	142.29	8.78	74.29	NR

*Review included 10 studies. Costs were updated to 2022 US dollars by using the healthcare component of the Personal Consumption Expenditure Price Index (*18*). NR, not reported; TST, tuberculin skin test. †Labor time converted to labor cost by using the corresponding year's region-specific hourly wage for registered nurses from the US Bureau of Labor Statistics (*13*). ‡Included transportation cost.

After applying the percentage of contacts who received each type of test (e.g., in 2022, 37.6% of patients received TSTs, 54.2% of patients received QFT tests, and 8.3% received T-SPOT tests) (*22*), we applied the costs of testing for TST ($15.95) and the 2022 Medicare reimbursement rates for QFT tests ($61.89) and T-SPOT tests ($100.00) to calculate a weighted average testing cost of $47.21. Finally, in 2022, 13.2% of contacts tested positive for TB and were referred for a chest radiograph (*19*) at a cost of $43.79 per patient, which yielded an average chest radiograph cost of $5.77 per contact. Therefore, adding testing and chest radiograph costs to labor costs yielded a total contact tracing cost per contact of $228.93 (range $132.95–$346.49).

### National Cost of Contact Investigations

In 2022, jurisdictions across the United States reported conducting contact investigations for 33,576 contacts of persons with sputum smear–positive TB and 9,830 contacts of persons with sputum smear–negative, culture-positive TB (Appendix Table 1) (*19*). When extrapolating those costs to the US population, the estimated total US cost of contact investigations was $9.94 (range $5.77–$15.04) million. When totaled over the 10-year period (2013–2022), estimated contact investigation cost for health departments was $137.36 (range $75.61–$212.99) million (Appendix).

## Discussion

Contact investigation is an essential part of the public health strategy for TB care and prevention and is inherently labor intensive at the local level. We identified a relatively small number of published studies compared with the large national expenses that we estimated. Therefore, more information about contact investigation costs could enable better treatment and prevention planning and elucidate how those costs vary.

Our analysis projected a substantial range ($80–$294) in labor cost per contact investigation. We generally were not able to fully account for differences in costs between studies. However, community settings had the greatest cost per contact investigation; those higher costs could be associated with greater travel and communication costs. Differences in costs could also be attributable to heterogeneity in the type of contact (e.g., household contacts vs. close contacts), which in turn could lead to differences in inherent time spent per contact investigation. Furthermore, although this analysis accounted for costs associated with settings and labor, it is also possible that contact investigations with similar time commitments conducted by different agencies would expend greater (or less) resources simply on the basis of the locality and labor combination used. Differences in costs by locality also likely contribute to the labor cost uncertainty and, thus, the wide range in overall national costs. More information about specific costs expended by different US states or localities could help reduce the uncertainty associated with labor cost estimates.

The first limitation of our study is that some reports included costs not fully separable from overall contact investigation costs. For instance, 1 study included transportation costs; however, those transportation costs were not itemized and not separable from total costs (*29*). Similarly, not all studies delineated the type of staff conducting contact investigations, which could lead to differences in cost because of different compensation levels. Second, we did not account for surveillance costs, overhead, or other costs (e.g., telephone, computer, internet, and building costs), which could increase the estimated costs of contact investigation. Third, 4 of the 10 reports identified in this study were published before 2010, and more recent changes in TB testing and treatment could affect cost. For example, the increased use of IGRAs, which is more specific for *M. tuberculosis* infection, could reduce total contact investigation costs; fewer resources would be needed to address false-positive results that might be obtained from the less-specific TST (*6*). Nevertheless, the mean estimated cost per contact investigation for studies published after 2009 (n = 6) increased slightly from the overall mean to $196.80; however, that cost was still within the overall labor cost uncertainty interval. Recent improvements in telecommunication technologies (e.g., use of smart phones) also lowered communication costs that could affect contact investigation methods and costs. Finally, reported studies did not delineate the number of contacts used to estimate labor costs, and only 1 study provided a range ($0–$500) around the mean estimate of contact investigation costs (*29*). For this reason, we were not able to weight cost estimates by sample size or adjust for uncertainty across study estimates, which implies that users of those results should carefully consider the wide uncertainty range in addition to the mean estimate. Furthermore, our annual estimates of the proportion of patients tested by using TSTs or IGRA were derived from a sample representative of privately insured persons; estimates might differ among other types of insured populations.

Labor costs associated with contact investigation were 77% ($175.94) of the estimated total costs ($228.93) of contact investigation; contact investigations include labor costs for time spent eliciting and reaching out to contacts, as well as for diagnostic test costs and costs associated with chest radiographs. Costs associated with TB testing can sometimes be reimbursed or paid for by insurance, if the patient is insured. However, TB testing has a necessary preliminary step, which is identifying contacts who need testing by public health personnel and is often not accounted for nor reimbursable, potentially leading to underinvestment. Another key feature of contact investigation is that not all investigations are identical, and contact investigation effects and costs can vary across jurisdictions (*31*). We attempted to incorporate those differences by using nonparametric methods to estimate CIs. This method enabled skewed data to be reflected in asymmetric CIs, thereby enabling study heterogeneity to be reflected in cost ranges.

Our analyses only estimated the cost of contact investigation. A related study estimated a cost per gained quality-adjusted life year of $27,800 over a 10-year period (*8*). That cost per quality-adjusted life year estimate is lower than in other studies examining the cost-effectiveness of targeted testing and treatment of persons with latent TB infection, which typically ranged from $80,000 to $150,000 per gained quality-adjusted life year (*32*–*34*). The lower estimate implies that outbreak investigations might be one of the most cost-effective ways to prevent TB. Independent of cost-effectiveness, contact investigations are necessary to prevent the need for ongoing TB treatment and avert TB disease costs that are paid by patients, healthcare providers, and federal, state, and local health agencies.

In conclusion, we provide a national estimate of contact investigation costs for TB in the United States. Contact investigation, a core public health activity, directly identifies persons infected with TB and drives focused public health action to prevent TB-associated illness and death. Although contact investigations are essential to prevent TB, benefits only accrue when exposed contacts are identified and evaluated and when TB disease or *M. tuberculosis* infections are fully diagnosed and resolved through treatment.

Appendix Additional information for systematic review of contact investigation costs for tuberculosis, United States.
