# Supplementary material for: Systematic Review of Contact Investigation Costs for Tuberculosis, United States
Source: Emerg Infect Dis. 2025 Jul;31(7):1287–93. doi: 10.3201/eid3107.241827 (PMC12205448; doi:10.3201/eid3107.241827)
Supplement: Appendix — Additional information for systematic review of contact investigation costs for tuberculosis, United States. [file 24-1827-Techapp-s1.pdf]

Article DOI: https://doi.org/10.3201/eid3107.241827

EID cannot ensure accessibility for supplementary materials supplied by authors. Readers who have difficulty accessing supplementary content should contact the authors for assistance.

Systematic Review of Contact Investigation Costs for Tuberculosis, United States

Appendix

Additional Methods

Estimation of National Contact Investigation Costs

To estimate national contact investigation costs, we used the following equation:

$$\text{Total Cost}_t = N_t \times (LC + TST_{p,t} \times TST_c + QFT_{p,t} \times QFT_c + TSPOT_{p,t} \times TSPOT_c + PTP_t \times CXR_c)$$

Where *t* denotes a year during 2013–2022, *p* denotes proportion of contacts, and *c* denotes unit cost. N_t denotes total contacts (smear positive, culture positive and smear negative, culture positive) elicited as reported by the ARPE report for year *t (1–3)*. LC denotes the mean labor cost per contact for contact investigation from the systematic review ($175.94 [range $79.97–$293.51]). $TST_{p,t}$, $QFT_{p,t}$, and $TSPOT_{p,t}$ denote the proportion of tuberculin skin tests (TST), QFT, and TSPOT tests given to contacts in year *t* (*4*). TST_c, QFT_c, and $TSPOT_{c,t}$ denote the cost per test for a TST ($14.16), QFT test ($61.89), and TSPOT assay ($100) in 2022 (*5*). PTP_t denotes the proportion of contacts that tested positive in year *t* (*1–3*), and CXR_c denotes the cost of chest radiograph ($43.79). Using the above formula, we computed total contact investigation costs for each year during 2013–2022 and summed the yearly costs to obtain 10-year costs. We also used a 95% confidence estimate for labor costs to generate a CI for all estimates.

Community Guide Quality Rating

According to the Community Guide, studies were judged as having “good,” “fair,” or “limited” quality of execution; quality of execution is defined through the Community Guide’s assessment framework (*6*). Studies with 0–1, 2–4, and ≥5 limitations were categorized as having good, fair, and limited execution, respectively. Of the included studies, 9 were judged to be good

quality (*7–15*), and 1 was fair quality (*16*). Because none of the studies were judged to have "limited execution," all studies were included in the review.

Appendix Table 1. TB tests among privately insured patients and total numbers of contacts elicited, United States, 2013–2022*

	% Patients tested†			Total contacts‡		
Year	TST	QFT	T-SPOT	% Contacts with positive test	No. smear positive, culture positive index case	No. smear negative, culture positive index case
2013	86.7	12.5	0.9	14.7	69,063	16,063
2014	81.9	17.1	1.0	13.2	65,590	14,847
2015	76.1	22.5	1.6	11.8	64,509	17,680
2016	69.5	28.5	2.2	11.8	59,721	13,914
2017	64.1	33.4	2.6	11.8	51,392	14,100
2018	59.0	38.6	2.5	11.1	53,783	13,589
2019	52.1	42.0	6.2	10.5	51,419	12,679
2020	46.0	46.2	8.2	11.6	35,918	8,829
2021	40.7	51.1	8.5	14.6	28,736	8,128
2022	37.6	54.2	8.3	13.2	33,576	9,830

*QFT, QuantiFERON-TB Gold blood assay; T-SPOT, tuberculosis antigen immunospot assay; TST, tuberculin skin test.
†Analysis of Truven Health MarketScan Research Databases (2013–2022) (https://marketscan.truvenhealth.com/marketscanportal).
‡Contact data from 2017–2022 Contact Investigation Report (ARPE Data, Division of Tuberculosis Elimination, Centers for Disease Control and Prevention, Atlanta, Georgia (cited October 9, 2024).

Appendix Table 2. Search strategy used for the review

Database	Strategy
Medline (OVID) 1946–	Tuberculosis OR TB OR LTBI AND (outbreak* ADJ5 investigat*) OR (contact* ADJ5 trac*) OR (contact* ADJ5 investigat*) OR (epidemiolog* ADJ5 activit*) OR (epidemiolog* ADJ5 investigat*) OR (epidemiolog* ADJ5 link*) OR (population ADJ5 surve*) OR public health AND(cost* OR economic* OR expense* OR expenditure* OR financ* OR fund* OR resource* OR surveillance OR infrastructure OR staff OR personnel OR capital OR dollars OR spend* OR capacity building OR grant* OR investment* OR ec.fs) AND Exp United States/ OR United States OR USA OR Alabama OR Alaska OR Arizona OR Arkansas OR California OR Colorado OR Connecticut OR Delaware OR Florida OR Georgia OR Hawaii OR Idaho OR Illinois OR Indiana OR Iowa OR Kansas OR Kentucky OR Louisiana OR Maine OR Maryland OR Massachusetts OR Michigan OR Minnesota OR Mississippi OR Missouri OR Montana OR Nebraska OR Nevada OR New Hampshire OR New Jersey OR New Mexico OR New York OR North Carolina OR North Dakota OR Ohio OR Oklahoma OR Oregon OR Pennsylvania OR Rhode Island OR South Carolina OR South Dakota OR Tennessee OR Texas OR Utah OR Vermont OR Virginia OR Washington OR West Virginia OR Wisconsin OR Wyoming OR New England OR Mid-Atlantic OR (U.S. ADJ3 cities) OR United States.gi; Limit English; Abstracts Available; Limit to yr = 1990–2024
Embase (OVID) 1974–	Tuberculosis OR TB OR LTBI AND (outbreak* ADJ5 investigat*) OR (contact* ADJ5 trac*) OR (contact* ADJ5 investigat*) OR (epidemiolog* ADJ5 activit*) OR (epidemiolog* ADJ5 investigat*) OR (epidemiolog* ADJ5 link*) OR (population ADJ5 surve*) OR public health AND (cost* OR economic* OR expense* OR expenditure* OR financ* OR fund* OR resource* OR surveillance OR infrastructure OR staff OR personnel OR capital OR dollars OR spend* OR capacity building OR grant* OR investment*) AND Exp United States/ OR United States OR USA OR Alabama OR Alaska OR Arizona OR Arkansas OR California OR Colorado OR Connecticut OR Delaware OR Florida OR Georgia OR Hawaii OR Idaho OR Illinois OR Indiana OR Iowa OR Kansas OR Kentucky OR Louisiana OR Maine OR Maryland OR Massachusetts OR Michigan OR Minnesota OR Mississippi OR Missouri OR Montana OR Nebraska OR Nevada OR New Hampshire OR New Jersey OR New Mexico OR New York OR North Carolina OR North Dakota OR Ohio OR Oklahoma OR Oregon OR Pennsylvania OR Rhode Island OR South Carolina OR South Dakota OR Tennessee OR Texas OR Utah OR Vermont OR Virginia OR Washington OR West Virginia OR Wisconsin OR Wyoming OR New England OR Mid-Atlantic OR (U.S. ADJ3 cities); Limit English; Abstracts Available; Limit to yr = 1990–2024
CINAHL (EBSCOHost)	Tuberculosis OR TB OR LTBI AND (outbreak* N5 investigat*) OR (contact* N5 trac*) OR (contact* N5 investigat*) OR (epidemiolog* N5 activit*) OR (epidemiolog* N5 investigat*) OR (epidemiolog* N5 link*) OR (population N5 surve*) OR "public health" AND (cost* OR economic* OR expense* OR expenditure* OR financ* OR fund* OR resource* OR surveillance OR infrastructure OR staff OR personnel OR capital OR dollars OR spend* OR capacity building OR grant* OR investment*) AND "United States" OR USA OR Alabama OR Alaska OR Arizona OR Arkansas OR California OR Colorado OR Connecticut OR Delaware OR Florida OR Georgia OR Hawaii OR Idaho OR Illinois OR Indiana OR Iowa OR Kansas OR Kentucky OR Louisiana OR Maine OR Maryland OR Massachusetts OR Michigan OR Minnesota OR Mississippi OR Missouri OR Montana OR Nebraska OR Nevada OR "New Hampshire" OR "New Jersey" OR "New Mexico" OR "New York" OR "North Carolina" OR "North Dakota" OR Ohio OR Oklahoma OR Oregon OR Pennsylvania OR "Rhode Island" OR "South Carolina" OR "South Dakota" OR Tennessee OR Texas OR Utah OR Vermont OR Virginia OR Washington OR "West Virginia" OR Wisconsin OR Wyoming OR "New England" OR Mid-Atlantic OR (U.S. N3 cities); Limit English; Abstracts Available; Limit to yr = 1990–2024

Database	Strategy
Scopus	TITLE-ABS-KEY(Tuberculosis OR TB OR LTBI) AND TITLE-ABS-KEY((outbreak* W/5 investigat*) OR (contact* W/5 trac*) OR (contact* W/5 investigat*) OR (epidemiolog* W/5 activit*) OR (epidemiolog* W/5 investigat*) OR (epidemiolog* W/5 link*) OR (population W/5 surve*) OR “public health”) AND TITLE-ABS-KEY(cost* OR economic* OR expense* OR expenditure* OR financ* OR fund* OR resource* OR surveillance OR infrastructure OR staff OR personnel OR capital OR dollars OR spend* OR “capacity building” OR grant* OR investment*) AND TITLE-ABS-KEY(“United States” OR USA OR Alabama OR Alaska OR Arizona OR Arkansas OR California OR Colorado OR Connecticut OR Delaware OR Florida OR Georgia OR Hawaii OR Idaho OR Illinois OR Indiana OR Iowa OR Kansas OR Kentucky OR Louisiana OR Maine OR Maryland OR Massachusetts OR Michigan OR Minnesota OR Mississippi OR Missouri OR Montana OR Nebraska OR Nevada OR “New Hampshire” OR “New Jersey” OR “New Mexico” OR “New York” OR “North Carolina” OR “North Dakota” OR Ohio OR Oklahoma OR Oregon OR Pennsylvania OR “Rhode Island” OR “South Carolina” OR “South Dakota” OR Tennessee OR Texas OR Utah OR Vermont OR Virginia OR Washington OR “West Virginia” OR Wisconsin OR Wyoming OR “New England” OR Mid-Atlantic OR (U.S. W/3 cities)) AND NOT INDEX(medline); Limit English; Abstracts Available; Limit to yr = 1990–2024

References

1. Centers for Disease Control and Prevention. 2022 ARPE contact investigation report. October 16, 2024 [cited 2025 Jun 4]. . https://www.cdc.gov/tb-data/arpe-reports/2022-contact-investigations.html

2. Centers for Disease Control and Prevention. 2020 Contact investigation report (ARPE data) [cited 2025 Jun 4]. https://archive.cdc.gov/www_cdc_gov/tb/programs/evaluation/arpe-data.htm

3. Centers for Disease Control and Prevention. 2017 Contact Investigation Report (ARPE Data). Atlanta, (GA): Centers for Disease Control and Prevention; 2020.

4. Beeler Asay GR, Bohm MK. Tuberculosis diagnostic test use in the United States privately insured population, 2013–2022. In: Joint Conference of The Union–North American Region and the National TB Coalition of America; Baltimore, MD, USA; 2024 Apr 16-19. Abstract 2 (poster session, Scientific Reporting/Diagnostics for TB Disease and Infection, Apr 18).

5. Centers for Medicare and Medicaid Services. Clinical laboratory fee schedule. 2022 [cited 2024 Nov 19]. https://www.cms.gov/medicare/payment/fee-schedules/clinical-laboratory-fee-schedule-clfs

6. Community Preventive Services Task Force. Methods manual for community guide systematic reviews [cited 2025 Mar 21]. https://www.thecommunityguide.org/methods-manual

7. Shiau R, Holmen J, Chitnis AS. Public health expenditures and clinical and social complexity of tuberculosis cases—Alameda County, California, July–December 2017. J Public Health Manag Pract. 2022;28:188–98. PubMed https://doi.org/10.1097/PHH.0000000000001356

8. Kelly AM, D’Agostino JF, Andrada LV, Liu J, Larson E. Delayed tuberculosis diagnosis and costs of contact investigations for hospital exposure: New York City, 2010–2014. Am J Infect Control. 2017;45:483–6. PubMed https://doi.org/10.1016/j.ajic.2016.12.017

9. Kerr CM, Savage GT. Managing exposure to tuberculosis in the PACU: CDC guidelines and cost analysis. J Perianesth Nurs. 1996;11:143–6. PubMed https://doi.org/10.1016/S1089-9472(96)90003-9

10. Miller TL, McNabb SJ, Hilsenrath P, Pasipanodya J, Drewyer G, Weis SE. The societal cost of tuberculosis: Tarrant County, Texas, 2002. Ann Epidemiol. 2010;20:1–7. PubMed https://doi.org/10.1016/j.annepidem.2009.09.004

11. Pisu M, Gerald J, Shamiyeh JE, Bailey WC, Gerald LB. Targeted tuberculosis contact investigation saves money without sacrificing health. J Public Health Manag Pract. 2009;15:319–27. PubMed https://doi.org/10.1097/PHH.0b013e31819c3ef2

12. Centers for Disease Control and Prevention. Transmission of *Mycobacterium tuberculosis* in a high school and school-based supervision of an isoniazid-rifapentine regimen for preventing tuberculosis—Colorado, 2011–2012. MMWR Morb Mortal Wkly Rep. 2013;62:805–9. PubMed

13. Brown RE, Miller B, Taylor WR, Palmer C, Bosco L, Nicola RM, et al. Health-care expenditures for tuberculosis in the United States. Arch Intern Med. 1995;155:1595–600. PubMed https://doi.org/10.1001/archinte.1995.00430150057006

14. Cruz AT, Medina D, Whaley EM, Ware KM, Koy TH, Starke JR. Tuberculosis among families of children with suspected tuberculosis and employees at a children's hospital. Infect Control Hosp Epidemiol. 2011;32:188–90. PubMed https://doi.org/10.1086/657940

15. Park PH, Holland DP, Wade A, Goswami ND, Bissette D, Stout JE. Public health costs for tuberculosis suspects in Wake County, North Carolina, United States. Int J Tuberc Lung Dis. 2013;17:759–63. PubMed https://doi.org/10.5588/ijtld.12.0739

16. Sprinson JE, Flood J, Fan CS, Shaw TA, Pascopella L, Young JA, et al. Evaluation of tuberculosis contact investigations in California. Int J Tuberc Lung Dis. 2003;7:S363–8. PubMed
